# The pitfalls of positional dizziness: Not everything is crystal

**DOI:** 10.1016/j.bjorl.2025.101575

**Published:** 2025-03-22

**Authors:** Lucas Resende Lucinda Mangia, Raquel Mezzalira, Roseli Saraiva Moreira Bittar

**Affiliations:** a Universidade Federal do Paraná (UFPR), Curitiba, PR, Brazil; b Universidade Estadual de Campinas (UNICAMP), Campinas, SP, Brazil; c Universidade de São Paulo (USP), São Paulo, SP, Brazil

Recently, a large number of patients with dizziness have been misdiagnosed. These mistakes lead patients to a pilgrimage in search of help. Along their rocky path, they are subjected to unnecessary therapies and dangerous delays in their diagnoses, if not disastrous consequences. These events are likely behind an increase in the burden related to vestibular diseases and a rise in health costs.

Positional vestibular complaints are notably prevalent among these cases. Therefore, it is important to recognize that consistent flaws have been occurring in the assessment of these patients. Here, diagnostic failures are mainly the result of oversimplification of clinical investigation. Frequently, patients with positional dizziness do not undergo a systematic evaluation. Also, those who treat them often ignore that a reasonable share of cases is caused by conditions other than Benign Paroxysmal Positional Vertigo (BPPV).

Despite being the most common cause of positional dizziness, BPPV is not the only one. Hence, the diagnosis of BPPV must be preceded by compatible clinical findings.^1^ In other words, appropriate nystagmus arising from stimulation and/or inhibition of the affected semicircular canals ought to be found by properly performing the provocative maneuvers. This nystagmus has well-defined features and behaviors, which mirror their peripheral origin and mechanical nature. The popularization of repositioning maneuvers among healthcare professionals has given BPPV patients easier access to effective treatments. However, their use should be encouraged only after proper evaluation. Taking every positional dizziness as BPPV is a dangerous generalization that is not supported by scientific evidence. Yet, the resolution of BPPV after appropriate repositioning maneuvers is the rule. Repeating them indefinitely, without considering alternative diagnoses, is not recommended. Pitfalls while treating individuals with positional dizziness could be prevented by knowing the differential diagnoses and following a careful approach to every single case.

A wide variety of mechanisms might underlie positional dizziness and nystagmus. Some of them carry substantial risks if their diagnosis is neglected. The complete neurotological physical exam and the meticulous evaluation of nystagmus, ideally with the aid of equipment allowing removal of ocular fixation, are non-negotiable key elements in the study of positional disorders.^1^, ^2^, ^3^

Central Positional Nystagmus (CPN) accounts for 12% of positional nystagmus and is associated with various structural or functional lesions of the cerebellum and/or brainstem.^2^ It shows unusual features and should prompt a comprehensive etiological investigation.^2^, ^3^ Pure vertical nystagmus, downbeat in particular, apogeotropic nystagmus resistant to appropriate maneuvers, and multidirectional nystagmus are atypical findings that should raise suspicion for CPN. During positional testing, nystagmus without latency, persistent low-velocity nystagmus or the absence of concomitant symptoms are also frequent red flags. Individuals with positional nystagmus and increased cardiovascular risk, visual and/or auditory complaints, or oculomotor abnormalities should be carefully evaluated, particularly in acute settings.

Dizziness and positional nystagmus can often be attributed to conditions with increasing recognition in Neurotology, but still poorly described in the literature. That could be the case of cervical proprioceptive dysfunctions or metabolic disorders, especially those related to glycemic control.^4^, ^5^ In the former case, clinical findings would occur due to the mismatch between inputs generated in the posterior labyrinth and those coming from anomalous cervical afferents during positional changes.^4^ In the latter, endolymphatic chemical and density alterations would lead to abnormal cupular function.^5^ Importantly, these hypotheses should be cogitated only after a comprehensive assessment and full consideration of other possibilities.

Asymmetries in vestibular tone of any origin might explain positional dizziness. In these cases, insufficient vestibular compensation could account for suboptimal vestibulo-ocular reflex gain following positional changes. Thus, healthcare professionals should include positional symptoms as part of the plethora of complaints possibly related to decompensated vestibular diseases. Even patients with confirmed BPPV can sometimes require an extended evaluation and specialized care. Cases with recurrent outbreaks or associated with other vestibular disorders are good examples.

As positional dizziness could be tricky, caution is warranted when using shortcuts in their approach, such as prescribing treatments without proper evaluation. For instance, the indiscriminate use of home maneuvers might carry risks. Nor is there solid scientific ground for the unsystematic use of repositioning maneuvers in atypical scenarios. These include positional symptoms induced by the diagnostic maneuvers but without concurrent nystagmus or cases with positional nystagmus with abnormal features.

Healthcare professionals who treat positional dizziness must be familiar not only with BPPV, but also with the differential diagnoses. It is essential to proceed with prudence in less typical cases, and avoid depriving the patient of specialized assessment and management. The clinical approach to positional dizziness, similar to other vestibular symptoms, must be thorough and targeted.

## Declaration of competing interest

The authors declare no conflicts of interest.
